# Neuropathology of an unselected forensic autopsy cohort: Tampere Sudden Death Study

**DOI:** 10.1002/alz70861_108057

**Published:** 2025-12-23

**Authors:** Eloise H Kok

**Affiliations:** ^1^ Tampere University Hospital (TAYS), Tampere, Pirkanmaa, Finland; Tampere University, Tampere, Pirkanmaa Finland

## Abstract

**Background:**

Understanding the prevalence of brain pathology markers in an unselected population will provide the best indications of when therapeutic avenues need to be initiated, as most likely the earliest stages of accumulation will be the most effective timepoint to administer treatments. It still remains unknown how the main brain changes associated with dementia actually impact disease progression, and whether they are causative or simply bystanders.

**Method:**

The Tampere Sudden Death Study (TSDS) consists of 700 forensic autopsied individuals, aged 16‐97 years, who died suddenly outside of hospitals within an area in south eastern Finland. Immunohistochemical staining of the main pathological markers associated with dementia disorders, including amyloid beta, tau, alpha‐synuclein, TDP‐43, and small vessel disease parameters have been or are being processed. Measurements and grading of each of these pathologies provide a score for each pathology, which can be complemented with genetic or proteomic analyses.

**Result:**

Findings of alpha‐synuclein have already been published, with tau and amyloid beta scoring almost complete. TDP‐43 and small vessel disease assessments require new collaborators to complete!

**Conclusion:**

The unique TSDS cohort has already proven valuable in showing that alpha‐synuclein pathology occurs already a decade before previously thought. When the complete pathological work up of these samples is finalised, we will be able to better understand the prevalence of these markers in a non‐selected population. Identifying which pathologies co‐occur and potential impacts on each other will assist in the development of effective treatments against these devastating brain diseases.

